# Impact of psychiatric disorders on pregnancy and its management. A French retrospective cohort study

**DOI:** 10.1192/j.eurpsy.2023.1353

**Published:** 2023-07-19

**Authors:** E. Briaud, D. Doolub, S. Guinot, J. Deparis, N. Jaafari

**Affiliations:** 1 Ecole de Sages-Femmes, Faculté de médecine et de pharmacie de Poitiers, CHU de Poitiers; 2 Experimental psychology, CNRS, Université de Poitiers, Université de Tours, CeRCA, Poitiers, France; 3 Neuromodulation and Adult Psychiatry, Pierre Deniker Research Center; 4 Ecole de Sages-Femmes, Faculté de médecine et de Pharmacie de Poitiers, Centre Hospitalier Universitaire de Poitiers, Poitiers, France

## Abstract

**Introduction:**

Every pregnancy and birth is unique, and despite this, few studies exist on the condition of pregnant women with mental disorders.

**Objectives:**

This study analyzes the impact of prevalent mental disorders in pregnant women to determine which clinical or socio-demographic characteristics significantly impact pregnancy.

**Methods:**

This retrospective and naturalistic cohort study is based on the medical records of 99 patients managed in a university hospital. All patients had an ICD-10 mental disorder (psychiatric and/or substance use disorders) diagnosed before pregnancy.

**Results:**

Only 24.2% of the pregnant women had no adverse outcomes throughout pregnancy, labor, and delivery. The remaining mothers had violence issues, and mothers with psychotrauma were likelier to have stillborn babies. Pregnant women with mental disorders were less keen to screen for Down’s syndrome and more likely to have artificial delivery in case of comorbid drug addiction and alcohol use disorder (AUD). Anonymous birth and placement of newborns were related to substance abuse and pre-pregnancy AUD comorbidities or AUD alone before pregnancy. Besides, four clinical characteristics were found to be predictive of adverse pregnancy outcomes: young maternal age (*β* =-1,15,*p*<.03), late-term first contact with the maternity hospital (*β*=0.08, *p*<.02), advanced term of delivery (*β*=4.01, *p*<.03), and a history of psychiatric disorders associated with an AUD but without smoking before pregnancy (*β* =-1,07,*p*<.03). Despite all, pregnant women had a relatively sustained follow-up of their pregnancies.

**Conclusions:**

Mental disorders have a negative impact on pregnancy. More studies should be promoted to raise the attention of professionals to manage and improve women’s pregnancy and motherhood with psychiatric conditions.

**Disclosure of Interest:**

None Declared

